# Nutrient intake, serum lipids and iron status of colligiate rugby players

**DOI:** 10.1186/1550-2783-10-9

**Published:** 2013-02-13

**Authors:** Hiroyuki Imamura, Kazuhide Iide, Yoshitaka Yoshimura, Kenya Kumagai, Reika Oshikata, Keiko Miyahara, Kazuto Oda, Noriko Miyamoto, Anthony Nakazawa

**Affiliations:** 1 Faculty of Health Management, Department of Health and Nutrition, Nagasaki International University, 2825-7 Huis Ten Bosch, Sasebo-shi, Nagasaki, 859-3298, Japan; 2 Department of Physical Education, International Pacific University, 721 Kannonnji, Seto-cho, Higashi-ku, Okayama, 709-0863, Japan; 3 Department of Food and Nutrition, Beppu University, 82 Kitaishigaki, Beepu-shi, Oita, 874-8501, Japan; 4 Faculty of Human and Social Studies, Department of International Tourism, Nagasaki International University, 2825-7 Huis Ten Bosch, Sasebo-shi, Nagasaki, 859-3298, Japan; 5 Faculty of Life Sciences, Seika Women’s Junior College, 2-12-1 Minami Hachiman-cho, Hakata-ku, Fukuoka, 812-0886, Japan; 6 Faculty of Health and Welfare, Nishikyushu University, 4490-9 Ozaki, Kanzaki-machi, Kanzaki-shi, Saga, 842-8585, Japan; 7 Faculty of Cooperative Extension Service, University of Alaska Fairbanks, PO Box 210261, Anchorage, 99521, Alaska

**Keywords:** Athlete, Lipoprotein cholesterol, Apolipoprotein, Physical activity, Anemia, Iron depletion

## Abstract

**Background:**

There are two main playing positions in rugby (backs and forwards), which demonstrate different exercise patterns, roles, and physical characteristics. The purpose of this study was: 1) to collect baseline data on nutrient intake in order to advise the athletes about nutrition practices that might enhance performance, and 2) to compare serum lipids, lipoproteins, apolipoproteins (apo), lecithin:cholesterol acyltransferase (LCAT) activity, and iron status of forwards and backs.

**Methods:**

The sporting group was divided into 18 forwards and 16 backs and were compared with 26 sedentary controls. Dietary information was obtained with a food frequency questionnaire.

**Results:**

There were significant differences among the three groups. The forwards had the highest body weight, body mass index, percentage of body fat (calculated by sum of four skinfold thicknesses), as well as the highest lean body mass, followed by the backs and the control group. The mean carbohydrate intake was marginal and protein intake was lower than the respective recommended targets in all three groups. The mean intakes of calcium, magnesium, and vitamins A, B_1_, B_2_, and C were lower than the respective Japanese recommended dietary allowances or adequate dietary intakes for the rugby players. The forwards had significantly lower high-density lipoprotein cholesterol (HDL-C) and HDL_2_-C than the backs and had significantly higher apo B and LCAT activity than the controls. The backs showed significantly higher HDL-C, HDL_3_-C, low-density lipoprotein cholesterol, and apo A-I, and LCAT activity than the controls. Four forwards (22%), five backs (31%), and three controls (12%) had hemolysis. None of the rugby players had anemia or iron depletion.

**Conclusion:**

The findings of our study indicate that as the athletes increased their carbohydrate and protein intake, their performance and lean body mass increased. Further, to increase mineral and vitamin intakes, we recommended athletes increase their consumption of green and other vegetables, milk and dairy products, and fruits. The forwards showed more atherogenic lipid profiles than the backs, whereas the backs showed not only anti-atherogenic lipid profile, but also showed more atherogenic lipid profile relative to the control group. Additionally, our study showed none of the rugby players experienced anemia and/or iron depletion.

## Background

Rugby is a popular sport globally, with the International Rugby Board encompassing 92 national unions. Playing positions in rugby may be broadly classified as forwards and backs, which demonstrate different exercise patterns and roles. The forwards take part in scrums that involve physical impact and muscular performance, in addition to running and tackling. The backs display an exercise pattern focused on running and speed, in addition to some tackling [1].

Given the different demands placed on forwards and backs, physical characteristics differ between these positions. Generally the forwards have higher body fat than the backs, which may serve as a protective buffer in contact situations. The backs have lower body fat than the forwards, which may reflect the higher speed requirements for these players [1]. Lean subjects, in comparison with their counterparts, tend to show higher high-density-lipoprotein cholesterol (HDL-C) and lower low-density-lipoprotein cholesterol (LDL-C) [2,3]. It has been shown that low HDL-C concentrations and high LDL-C concentrations are associated with increased risk of coronary heart disease [4-6]. High physical activity is one of the factors shown to be associated with high HDL-C concentrations [7], which may explain, in part, the decreased risk of coronary heart disease in physically active people [8]. A 2002 study reported the lipid profile of rugby players [9] showed paradoxical decreases in HDL-C and apolipoprotein (apo) A-I in rugby players compared with those in control groups. However, this study only compared rugby players as a single group with a control group.

Because running and physical contact (such as tackling and scrumming) play an essential role in rugby training and matches, participating players have risk factors for iron depletion, which include hemolysis caused by repeated foot strikes and physical contact, as well as iron loss through gastrointestinal and urinary tracts, and sweating [10]. Regarding the occurrence of hemolysis, one study [11] reported on the iron status of rugby players. The results of the study showed continuous occurrence of hemolysis in the players. However, this study only compared rugby players as a single group with a control group.

Many of the studies on the lipid [6,12,13] and iron [14,15] status of athletes primarily examine their relative endurance activities, whereas the lipid and iron status of rugby players is less known. The purpose of this study of rugby players was: 1) to collect baseline data on nutrient intake in order to advise athletes about nutrition practices that might enhance performance, and 2) to compare serum lipids, lipoproteins, lecithin:cholesterol acyltransferase (LCAT) activity, and iron status of the forwards and backs.

## Methods

### Subjects

The sporting group consisted of 34 male rugby players who competed in the All Japan Collegiate Championship. They were divided into two groups, 18 forwards and 16 backs, and were compared with 26 sedentary controls. The players had maintained their training schedule, which consisted of aerobic and anaerobic exercises all year round (at least six days/week, two trainings/day, and two hours/day), and had played one match a week for more than 4 years. The mean (± SD) experiences of the forwards and backs were 5.6 ± 3.8 years and 6.5 ± 3.3 years, respectively. Because almost all participating university students belonged to sport clubs at their respective university, collegiate controls from three other universities were solicited for participation. They had been sedentary, except when taking a physical education class once a week, for at least 1 year. All data were obtained in June, which was considered representative of athletes’ physiological status during pre-season training. The subjects were all non-smokers and were not taking any drugs known to affect lipid and lipoprotein metabolism. The study protocol was approved by the ethics committee of the participating universities. Informed consent was obtained from each participant of this study.

### Measurements and dietary information

Body weight and height were measured with the subjects in underwear to the nearest 0.1 kg and 0.1 cm, respectively. The body mass index (BMI) was determined as weight/height^2^ (kg/m^2^). The biceps brachii, triceps brachii, subscapular, and suprailiac skinfold thicknesses were measured with a Harpenden caliper on the right side of the body with the subject in a standing position and are expressed as the mean of three consecutive measurements. The average of three measurements at each site was used to calculate the body density [16], percentage of body fat (%Fat), and lean body mass (LBM) [17].

All subjects were interviewed by experienced dietitians using a food frequency questionnaire (FFQ), which is based on 29 food groups and 10 types of cooking, for estimating the energy and nutrient intakes of each subject during the past one to two months [18]. From FFQ’s, the selected mean daily dietary and nutrient intakes were calculated according to the Tables of Japanese Foodstuff Composition [19]. Information on nutrient supplements and/or on diet was obtained via a self-administered questionnaire. The accuracy of the questionnaire was checked through individual interviews.

### Blood analysis

Physical exercise and beverages other than water were not allowed 36 h prior to the blood sampling. Subjects arrived at the laboratory by 0800 h. The temperature of the laboratory was set at 25°C. Fasting (12 h) blood samples were drawn from the antecubital vein after each subject had been seated quietly for at least 30 min. The samples were immediately stored in a cooler box, which was kept at 4°C until centrifugation was done in a refrigerated centrifuge at 4°C. Samples were analyzed by a local commercial laboratory (SRL Inc., Tokyo, Japan). All measurements were duplicated, and the results were reported within 2 weeks. Total cholesterol and triglycerides (TG) were analyzed by enzymatic methods. HDL-C was analyzed by direct assay with a selective inhibition method. HDL_2_-C and HDL_3_-C were analyzed by an ultracentrifugation method. LDL-C was analyzed by heparin and citrate precipitation method. LCAT activity was analyzed by a dipalmitoyl lecithin substrate method. Apo A-I and B were analyzed by a turbidimetric immunoassay method. Details of these methods and intra-assay and inter-assay coefficients of variation have been presented prior [20,21].

Red blood cells (RBC), hemoglobin (Hb), and hematocrit (Ht) were measured using an automated blood cell analyzer. Mean corpuscular volume (MCV) was calculated by Ht/RBC×10. Mean corpuscular hemoglobin (MCH) was calculated by Hb/RBC×10. Mean corpuscular hemoglobin concentration (MCHC) was calculated by Hb/Ht×100. Serum ferritin was measured by chemiluminescent enzyme immunoassay. Serum iron, total iron-binding capacity (TIBC), and unsaturated iron binding capacity were measured by a Nitroso-PSAP method. Serum transferrin was measured by a turbidimetric immunoassay method. Serum haptoglobin was measured by a nephelometry method. Percentage of saturated transferrin was calculated by serum iron/TIBC×100. Details of these methods for iron-related parameters have been presented elsewhere [22]. Anemia was defined as Hb level below 13 g/dl. Iron depletion was defined as ferritin level below 20 μg/L [23]. Hemolysis was defined as serum haptoglobin lower than the standard values reported by the commercial laboratory (SRL Inc., Tokyo, Japan).

### Statistical analysis

The SPSS statistical software 17.0J (Chicago, IL) was used to analyze the data. Descriptive statistics included means and SD. One-sample Kolmogorov-Smirnov test was performed to examine whether or not each parameter was normally distributed. Logarithmic transformation of TG was used to normalize the grossly skewed (p<0.05) distribution of this parameter. The mean differences among the three groups were determined by one-way analysis of variance. Scheffe’s test was used to identify specific significant differences when significant F values were identified. Two-sided p<0.05 was considered to be statistically significant.

## Results

The mean characteristics of the subjects are shown in Table 1. The forwards had significantly higher body weight, BMI, waist circumference, biceps brachii, subscapular, and suprailiac skinfold thicknesses, sum of 4 skinfold thicknesses, % fat, and LBM than the backs and control group. The backs had significantly higher body weight, BMI, triceps brachii, sum of 4 skinfold thicknesses, % fat, and LBM than the control group

**Table 1:** Anthropometric characterics of rugby players and controls

	**Forward**			**Backs**		**Controls**
	**(n=18)**			**(n=16)**		**(n=26)**
Age (yrs)	19.5 ± 0.9			19.5 ± 1.0		19.5 ± 1.1
Height (cm)	173.7 ± 5.9		†	171.2 ± 4.3		168.8 ± 6.9
Weight (kg)	87.3 ± 8.9	*	†	72.6 ± 7.4	†	58.5 ± 6.1
BMI (kg/m	28.9 ± 2.5	*	†	24.8 ± 2.0	†	20.5 ± 1.8
Waist (cm)	89.5 ± 9.5	*	†	78.7 ± 5.9		72.2 ± 5.3
Biceps brachii (mm)	8.9 ± 3.2	*	†	6.5 ± 3.6		4.6 ± 0.7
Triceps brachii (mm)	17.0 ± 4.0		†	13.7 ± 4.5	†	9.7 ± 3.6
Subscapular (mm)	19.3 ± 6.1	*	†	14.4 ± 5.1		11.0 ± 2.7
Suprailiac (mm)	20.9 ± 7.0	*	†	11.6 ± 6.1		8.3 ± 2.2
4 skin in fold (mm)	66.1 ± 18.0	*	†	46.3 ± 16.5	†	26.2 ± 8.1
% Fat	22.9 ± 4.1	*	†	18.8 ± 4.5	†	14.8 ± 2.4
LBM (kg)	68.3 ± 5.1	*	†	59.7 ± 5.1	†	50.4 ± 5.2

Values are the mean ± SD.

Abbreviations; *BMI*, body mass index; *% Fat*, Percentage of body fat; *LBM*, lean body mass.

*p < 0.05 vs Backs.

†p < 0.05 vs Controls.

The mean daily nutrient intakes are shown in Table 2. Among the rugby players, nine were occasionally taking protein and/or multi-vitamin and mineral supplements. Because the inclusion of supplements did not alter the results, the results are presented without the supplements. The forwards had significantly higher mean intakes of energy, fat, carbohydrate, saturated fat, polyunsaturated fat, potassium, calcium, magnesium, phosphorus, iron, vitamins B_1_ and B_2_ than the controls. The backs had significantly higher energy, carbohydrate, and magnesium intakes than the control group.

**Table 2:** Mean daily nutrient and dietary intakes of rugby players and controls

	**Forward**		**Backs**		**Controls**
	**(n=18)**		**(n=16)**		**(n=26)**
Energy (kcal)	3579 ± 848	†	2963 ± 111	†	2004 ± 427
Protein (g)	92.7 ± 22.3		79.9 ± 31.5		64.8 ± 15.7
Fat (g)	91.5 ± 25.0	†	77.2 ± 30.8		68.5 ± 19.7
Carbohydrate (g)	567.0 ± 160.1	†	457.4 ± 192.2	†	267.1 ± 62.5
Cholesterol (g)	403 ± 180		344 ± 249		339 ± 139
Saturated fat (g)	28.7 ± 9.1	†	25.2 ± 11.5		21.0 ± 6.3
Polyunsaturated fat (g)	17.3 ± 4.5	†	14.2 ± 5.1		13.6 ± 4.1
P/S ratio	0.63 ± 0.16		0.60 ± 0.13		0.67 ± 0.14
Potassium (mg)	2783 ± 850	†	2563 ± 906		1989 ± 474
Calcium (mg)	668 ± 268	†	554 ± 272		472 ± 147
Magnesium (mg)	311 ± 81	†	283 ± 91	†	209 ± 48
Phosphorus (mg)	1369 ± 357	†	1165 ± 446		937 ± 211
Iron (mg)	8.7 ± 2.9	†	7.2 ± 2.8		6.3 ± 1.7
V.A (?gRE)	526 ± 247		428 ± 239		411 ± 128
V.B1 mg/1000kcal	0.37 ± 0.12	†	0.31 ± 0.11		0.25 ± 0.06
V.B2 mg/1000kcal	0.40 ± 0.14	†	0.35 ± 0.16		0.29 ± 0.07
V.C (mg)	71 ± 42		56 ± 23		54 ± 19
Green vegetables (g)	37.2 ± 29.5		32.1 ± 38.0		59.2 ± 54.3
Other vegetables (g)	126.2 ± 51.4		95.5 ± 61.1		104.4 ± 59.2
Milk & dairy products (g)	233.9 ± 178.2		173.4 ± 173.5		145.0 ± 129.2
Fruits (g)	27.4 ± 50.5		25.6 ± 49.9		21.1 ± 26.6
Alchol (g)	1.95 ± 3.62		3.83 ± 3.99		1.43 ± 3.38

Values are the mean ± SD.

Abbreviations; *P/S*, polyunsaturated fat/saturated fat ratio; *V*, vitamin.

†p < 0.05 vs Controls.

The micronutrient intakes expressed as percentages of the Japanese dietary allowances (RDAs) or adequate dietary intakes (ADIs) are shown in Table 3. The mean intakes of calcium, magnesium, and vitamins A, B_1_, B_2_, and C were lower than the respective Japanese RDAs or ADIs in the rugby players. The mean intake of iron was above RDA in the forwards, whereas it was below in the backs. All micronutrient intakes were lower than the respective RDAs or ADIs in the control group.

**Table 3:** Micronutrient intakes expressed as percentages of the recommended dietary allowances (RDAs), and adequate dietary intakes (ADIs)

			**Forwarded (n=18)**	**Backs (n=16)**	**Controls (n=26)**
			**%**	**%**	**%**
Potassium (mg)	ADI	2000	139.2 ± 42.5	128.2 ± 45.3	99.4 ± 23.7
Calcium (mg)	ADI	900	74.3 ± 29.8	61.5 ± 30.2	52.4 ± 16.3
Magnesium (mg)	RDA	340	91.6 ± 23.8	83.4 ± 26.8	61.4 ± 14.1
Phosphorus (mg)	ADI	1050	130.4 ± 34.0	110.9 ± 42.5	89.2 ± 20.1
Iron (mg)	RDA	7.5	116.1 ± 39.1	96.4 ± 37.6	83.9 ± 23.1
V.A (?gRE)	RDA	750	70.1 ± 32.9	57.0 ± 31.9	54.7 ± 17.1
V.B_1_ mg/ 1000kca	RDA	0.54	68.3 ± 22.5	57.1 ± 20.8	46.1 ± 11.1
V.B_2_ mg/ 1000kcal	RDA	0.6	66.8 ± 23.7	58.0 ± 26.6	48.4 ± 12.1
V.C (mg)	RDA	100	71.4 ± 41.6	55.8 ± 23.3	53.9 ± 18.6

Values are the mean ± SD.

Abbreviations; *V*, vitamin.

The values of serum lipids, lipoproteins, apolipoproteins, and LCAT activity are shown in Table 4. The forwards had significantly lower HDL-C and HDL_2_-C than the backs and had significantly higher apo B and LCAT activity than the control group. The backs showed significantly higher HDL-C, HDL_3_-C, LDL-C, and apo A-I, and LCAT activity than the control group.

**Table 4:** Serum lipid, lipoprotein and apolipoprotein levels of rugby players and controls

	**Forward**		**Backs**		**Controls**
	**(n=18)**		**(n=16)**		**(n=26)**
HDL-C (mmol/l)	1.36 ± 0.18	*	1.61 ± 0.25	†	1.44 ± 0.23
HDL- C (mmol/l)	0.85 ± 0.15	*	1.05 ± 0.23		1.00 ± 0.21
HDL- C (mmol/l	0.51 ± 0.08		0.56 ± 0.07	†	0.46 ± 0.01
LDL-C (mmol/l)	2.74 ± 0.57		2.80 ± 0.85	†	2.27 ± 0.47
Lp (a) (mmol/l)	0.29 ± 0.32		0.31 ± 0.27		0.24 ± 0.25
TC (mmol/l)	4.37 ± 0.76		4.66 ± 0.97		3.99 ± 0.57
TG (mmol/l)	1.02 ± 0.56		0.87 ± 0.39		0.76 ± 0.23
logTG (mg/dl)	1.90 ± 0.23		1.85 ± 0.19		1.81 ± 0.13
Apo A- (mg/dl)	134.4 ± 18.8		149.6 ± 18.0	†	133.6 ± 17.5
Apo A-I (mg/dl)	30.3 ± 5.7		31.2 ± 4.8	†	26.9 ± 3.5
Apo B (mg/dl)	76.9 ± 15.9	†	78.1 ± 22.6		63.8 ± 12.7
LCAT activity (nmol/ml/h/37	83.3 ± 19.9	†	87.2 ± 20.1	†	65.5 ± 15.0

Values are the mean ± SD.

Abbreviations; *HDL-C*, high-density lipoprotein cholesterol; *LDL-C*, low-density lipoprotein cholesterol; *Lp*, lipoprotein; *Apo*, apolipoprotein; *LCAT* activity,lecitin:cholesterol. acyltransferase.

*p < 0.05 vs Backs.

†p < 0.05 vs Controls.

The hematological parameters are shown in Table 5. The forwards had significantly higher mean Ht, MCV, and lower MCHC than the control group. The backs had significantly higher transferring, TIBC, Ht, MCV, and significantly lower haptoglobin than the control group. Four forwards (22%), five backs (31%), and three controls (12%) had hemolysis (data not shown). None of the rugby players or controls had anemia. None of the rugby players had iron depletion, while one of the controls did.

**Table 5:** Hematological parameters of rugby players and controls

	**Forward**		**Backs**		**Controls**
	**(n=18)**		**(n=16)**		**(n=26)**
Ferritin (ng/ml)	73.4 ± 28.8		47.7 ± 17.6		72.0 ± 37.3
Transferrin (mg/dl)	262.8 ± 33.5		269.1 ± 28.5	†	243.8 ± 31.6
Serum iron (?g/dl)	17.6 ± 7.5		19.3 ± 5.9		19.3 ± 5.9
TIBC (?g/dl)	61.8 ± 7.4		63.6 ± 6.3	†	57.7 ± 7.0
UIBC (?g/dl)	44.2 ± 9.9		44.2 ± 7.8		38.4 ± 9.4
Red blood cell (×10000/?l)	503.3 ± 23.2		514.6 ± 19.0		515.7 ± 28.3
Hemoglobin (g/dl)	15.4 ± 0.8		15.8 ± 0.6		16.0 ± 0.9
Hematocrit (%)	50.7 ± 2.5	†	51.9 ± 2.3	†	48.6 ± 2.8
MCV (fl)	100.8 ± 4.3	†	100.9 ± 3.5	†	94.3 ± 3.0
MCH (pg)	30.7 ± 1.5		30.7 ± 0.8		31.0 ± 0.9
MCHC (%)	30.5 ± 0.8	†	30.4 ± 0.7	†	32.9 ± 0.6
Platelet (×10000/?l)	26.0 ± 4.0	*	21.8 ± 2.7		24.5 ± 3.8
Haptoglobin (mg/dl)	65.8 ± 36.9		51.9 ± 24.0	†	85.2 ± 41.5
Tf%	28.6 ± 12.2		30.5 ± 9.3		34.0 ± 11.1

Values are the mean ± SD.

Abbreviations; *TIBC*, total iron binding capacity; *UIBC*, unsaturated iron binding capacity; *MCV*= mean corpuscular volume,

*MCH*, mean conpuscular hemoglobin; *MCHC*, mean corpuscular hemoglobin concentration; Tf%, saturated transferrin.

*p < 0.05 vs Backs.

†p < 0.05 vs Controls.

## Discussion

### Nutrient intake

Lundy et al. [24] reported on the nutrient intake of Australian rugby players, in which the mean daily energy intakes of the forwards and backs were 4309±947 and 4142±822 kcal, respectively. In comparison with this previous study, the mean dietary energy intakes of the forwards (3579±848 kcal) and backs (2963±1119 kcal) were lower in the present study. To make valid comparison between the study by Lundy et al. [24] and the present study, we estimated the energy intakes in kcal kg^-1^ body weight in the study by Lundy et al. [24]. The estimated energy intakes of the forwards and backs were 43.8 and 48.4 kcal kg^-1^ body weight, respectively. In comparison with this study, the mean dietary energy intakes of the forwards (41.0 kcal kg^-1^ body weight) and backs (40.8 kcal·kg^-1^ body weight) were still lower in the present study. Thus, the divergence of results could be due to differences in not only the body weight, but also training status, skill levels, dietary differences, and/or ethnicity.

Our results indicate that adequate carbohydrate intake is important in rugby. The American College of Sports Medicine, the American Dietetic Association, and Dietetics of Canada (ACSM, ADA, & DC) [25] stated that a diet providing 500 to 600 g of carbohydrate (approximately 7 to 8 g·kg^-1^ BW for a 70-kg athlete) is adequate to sustain muscle glycogen stores during training and competition. According to these standards, the mean carbohydrate intakes of the forwards and backs (6.5±1.9 and 6.3±2.8 g·kg^-1^ body weight, respectively) in the present study were marginal.

ACSM, ADA, and DC [25] have recommended protein consumption of 1.2 to 1.4 g·kg^-1^·day^-1^ for endurance athletes and 1.6 to 1.7 g·kg^-1^·day^-1^ for resistance and strength-trained athletes. Because rugby is a high-intensity, intermittent activity, which requires aspects of both strength and endurance over a period of 80 min, we recommend 1.4 to 1.7 g·kg^-1^·day^-1^ of protein intake for rugby players. From this assumption, the mean protein intakes of the forwards and backs in the present study were lower than the recommendation (1.1±0.3 and 1.1±0.4 g·kg^-1^·day^-1^, respectively).

In the present study, the mean intakes of calcium, magnesium, and vitamins A, B_1_, B_2_, and C were lower than the respective Japanese RDAs or ADIs in the rugby players. Mean intakes below RDAs or ADIs in vitamins A, B_1_, and B_2_, iron, calcium, phosphorus, and/or magnesium have been reported in Japanese collegiate soccer players and karate practitioners [22,26]. To increase mineral and vitamin intakes, the Ministry of Health, Labor, and Welfare in Japan [27] recommends the consumption of 130 g of milk and dairy products, 120 g of green vegetables, and 230 g of other vegetables. In the rugby players, the mean intake of milk and dairy products was higher, but the intake of green and other vegetables was lower than the recommendations. The American and Canadian Dietetic Association’s [28] stated that the increased requirements for some minerals and vitamins during physical activity can be met by consuming a balanced high-carbohydrate, moderate-protein, low-fat diet.

One limitation of our study needs to be mentioned. In the present study, FFQ was used for estimating the energy and nutrient intakes of each subject during the past one to two months [18].

Because FFQ was developed to determine the most common food items for the population as a whole, its applicability for assessing the nutrient intakes of people whose eating patterns deviate considerably from those of the mainstream is limited. It is stated that FFQ may overestimate at low energy intakes and underestimate at high-energy intakes [29]. Thus, its applicability for assessing the nutrient intakes of rugby players regarding this study, especially players who show much higher or lower energy intake than the general population, may be limited. It has been stated that a 7-day dietary record increases the reliability of collected data [29]. However, in the present study, FFQ was chosen because it is much less burdensome than the 7-day dietary record, in consideration of the busy schedule of the subjects’ rugby training and academic studies. Even with this limitation taken into consideration, it is worthwhile to collect dietary assessments of these athletes because, as far as the authors are aware, this is the first study to examine serum lipids, lipoproteins, and iron status of rugby playing forwards and backs in Japan.

### Serum lipids, lipoproteins, apolipoproteins, and LCAT

One study [9] reported on the lipid profiles of rugby players, which showed a paradoxical decrease in HDL-C and apo A-I in the rugby players compared with those in the control group. However, this study only compared rugby players as a single group with controls and did not measure HDL-C subfractions. It has been shown that increased levels of HDL_2_-C, HDL_3_-C, and both subfractions were associated with decreased risk of myocardial infarction [30]. In the present study, we divided rugby players into forwards and backs and obtained different results. The forwards showed more atherogenic lipid profiles, such as significantly lower HDL-C and HDL_2_-C, than the backs, and significantly higher apo B than the control group. On the other hand, the backs showed not only anti-atherogenic lipid profile, such as significantly higher HDL-C, HDL_3_-C, and apo A-I, but also showed atherogenic lipid profile, such as significantly higher LDL-C, than the control group.

Proposed factors affecting blood lipid and lipoprotein concentrations include physical activity, body composition, dietary and nutrient intakes, cigarette smoking, and alcohol consumption [2,3,7,21,30]. In the present study, the subjects were all non-smokers. In addition, there were no significant differences among the three groups in terms of cholesterol, P/S ratio, intakes of yellow and green vegetables, other vegetables, and fruits, as well as alcohol consumption. Thus, influences of cigarette smoking, alcohol consumption, and these dietary and nutrient intakes appear to be limited. However, the cause of atherogenic and anti-atherogenic lipid profiles in rugby players could be multifactorial.

It has also been reported that BMI was positively correlated with LDL-C and TG, and was negatively correlated with HDL-C [2,3]. Excess caloric intake and saturated fatty acid intake raise serum LDL-C [31]. In addition, individuals consuming a high-carbohydrate diet tend to show lower HDL-C than those who consume a low-carbohydrate diet [32]. Thus, the increase in energy and carbohydrate intakes would be expected to raise LDL-C and lower HDL-C. In the present study, the forwards had significantly higher body weight, BMI, waist circumference, % fat, and LBM than the backs and control group. The backs had significantly higher body weight, BMI, % fat, and LBM than the control group. The forwards had significantly higher mean intakes of energy, fat, carbohydrate, and saturated fat than the control group. The backs had significantly higher energy and carbohydrate intakes than the control group. Thus, the causes of atherogenic and anti-atherogenic lipid and lipoprotein profiles of the forwards and backs could be due, at least in part, to the differences in physical characteristics and exercise demands and roles as mentioned above, nutrient intakes among the three groups, and/or a combination of these factors. In addition, differences in aerobic fitness such as maximal oxygen uptake could be another factor, although it is not measured in the present study. According to the review article by Duthie et al. [1], the backs typically possess greater levels of endurance fitness than the forwards.

Of the blood enzymes known to affect HDL metabolism, in the present study, the forwards and backs showed significantly higher LCAT activity than the control group. As far as we are aware, this is the first study to show increased LCAT activity in rugby players. Gupta et al. [33] also found increased LCAT activity in endurance athletes compared with that in controls. Frey et al. [34] found increased LCAT activity after a maximal aerobic stress test in both endurance-trained and sedentary groups. On the other hand, Brites et al. [35] compared LCAT activity between well-trained soccer players and controls and did not find a difference between the groups. Williams et al. [36] reported that a one-year running program did not significantly affect mean LCAT-mass concentrations. The divergent results obtained in these studies could be due to differences in age, physical activity, obesity, and/or other confounding factors as mentioned above.

### Hematological and iron status

Neither the rugby players nor the control group had anemia. The mean Hb in the forwards and backs (15.4±0.8 and 15.8±0.6 g/dl, respectively) was above the accepted standard value (13 g/dl) and was similar to that of the control group (16.0±0.9 g/dl). The forwards and backs showed significantly lower MCHC and higher MCV than the control group. The lower MCHC might be due to decreased iron availability for erythropoiesis and/or increased cell production. The higher MCV might be due to increased young red blood cells [37].

Iron deficiency reduces oxygen transport capacity and oxidative capacity at the cellular level, which develops rapidly or very slowly depending on the balance between iron intake and iron requirements [10]. For endurance-trained athletes, the total iron loss from feces, urine, and sweat has been estimated at about 1.75 g/dl [38]. The estimated basal iron loss and dietary iron absorption for Japanese men aged 18 to 29 years are 0.91 g/dl and 15%, respectively [27]. Although the dietary iron intakes of the forwards (8.7 g/dl × 0.15≒1.3 g/dl) and backs (7.2 g/dl × 0.15≒1.1 g/dl) would cover the basal iron loss, the calculated iron absorption for the forwards and backs appears to be lower than the estimated total iron loss for endurance-trained athletes [37].

Rugby players have risk factors for iron depletion, which include poor iron intake, hemolysis caused by repeated foot strikes and physical contact, iron loss through gastrointestinal and urinary tracts, and sweating. In the present study, the backs had significantly lower haptoglobin than the control group. However, only 22% of forwards and 31% of backs had hemolysis, which were much lower than the rate of hemolysis (71%) reported for soccer players [22]. Robinson et al. [39] suggested possible reasons for intravascular hemolysis as intramuscular destruction, osmotic stress, and membrane lipid peroxidation caused by free radicals released by active leukocytes. They also stated that intravascular hemolysis can even be regarded as a physiological means to provide heme and proteins for muscle growth. Serum haptoglobin binds the released Hb in order to prevent its urinary excretion. However, if hemolysis continues to persist throughout the season, haptoglobin may possibly be saturated with Hb, and Hb that could not bind to haptoglobin might be excreted with urine. Along with low dietary iron intake, this may lead to iron deficiency.

## Conclusions

Body mass is greater for the forwards than the backs. The mean carbohydrate intake was marginal and protein intake was lower than the respective recommended targets. Thus, we recommend athletes increase carbohydrate and protein intakes to increase performance and to develop LBM. The mean intakes of calcium, magnesium, and vitamins A, B_1_, B_2_, and C were lower than the respective Japanese RDAs or ADIs in the rugby players. The mean intake of iron was above RDA in the forwards, whereas it was below in the backs. To increase mineral and vitamin intakes, we recommend athletes increase consumptions of greens, other vegetables, milk, dairy products, and fruit. The forwards showed more atherogenic lipid profile than the backs, whereas the backs showed not only anti-atherogenic lipid profile, but also showed more atherogenic lipid profile relative to the control group. The causes of atherogenic and anti-atherogenic lipid profiles in rugby players could be multifactorial. None of the rugby players had anemia and iron depletion.

## Abbreviations

HDL-C: High-density-lipoprotein cholesterol; LDL-C: Lower low-density-lipoprotein cholesterol; TG: Triglycerides; Apo: Apolipoprotein; LCAT: Lecithin:cholesterol acyltransferase; FFQ: Food frequency questionnaire; BMI: Body mass index; LBM: Lean body mass; RDAs: Recommended dietary allowances; ADIs: Adequate dietary intakes; RBC: Red blood cells; Hb: Hemoglobin; Ht: Hematocrit; MCV: Mean corpuscular volume; MCH: Mean corpuscular hemoglobin; MCHC: Mean corpuscular hemoglobin concentration; TIBC: Total iron-binding capacity.

## Competing interests

The authors declare that they have no competing interests.

## Authors’ contributions

HI was the primary author of the manuscript. KI, YY, and KK designed the study and contributed to the interpretation. RO, KM, KO, and NM assessed dietary intake of the subjects and contributed to the data analysis and interpretation. AN contributed to the interpretation. All authors read and approved the final manuscript.
